# Achieving High Stability and Capacity in Micron‐Sized Conversion‐Type Iron Fluoride Li‐Metal Batteries

**DOI:** 10.1002/advs.202410114

**Published:** 2024-10-23

**Authors:** Chiwon Choi, Hyunmin Yoon, Seungyeop Kang, Dong Il Kim, John Hong, Minjeong Shin, Dong‐Joo Yoo, Minkyung Kim

**Affiliations:** ^1^ Department of Electronic Materials Engineering Kwangwoon University 60 Gwangun‐ro 1‐gil Nowon‐gu Seoul 01897 Republic of Korea; ^2^ School of Mechanical Engineering Korea University 145 Anam‐ro Seongbuk‐gu Seoul 02841 Republic of Korea; ^3^ Department of Materials Science and Engineering Kookmin University Seoul 02707 Republic of Korea; ^4^ School of Chemistry and Energy Center for NanoBio Applied Technology Sungshin Women's University 55 Dobong‐ro 76 ga‐gil Gangbuk‐gu Seoul 01133 Republic of Korea; ^5^ Center for NanoBio Applied Technology Sungshin Women's University 55, Dobong‐ro 76 ga‐gil Gangbuk‐gu Seoul 01133 Republic of Korea

**Keywords:** conversion‐type cathode, localized high concentration electrolytes for Li‐metal batteries, long‐term stability acheivement with micron‐sized FeF_2_, utilization of micron‐sized coversion materials

## Abstract

Iron fluoride, a conversion‐type cathode material with high energy density and low‐cost iron, holds promise for Li‐ion batteries but faces challenges in synthesis, conductivity, and cycling stability. This study addresses these issues by synthesizing micron‐sized iron‐fluoride using a simple solid‐state synthesis. Despite a large particle size, a high capacity of 571 mAh g^−1^ is achieved, which is attributed to the unique surface and internal pores within the iron‐fluoride particles, which provided a large surface area. This is the first study to demonstrate the feasibility of using large iron fluoride particles to enhance the energy density of the electrode and achieve an iron fluoride full cell with high capacity. Also, the cause of the capacity fading is investigated. Electrode delamination from the current collector, which is the main cause of capacity fading in early cycles, is resolved using a carbon‐coated aluminum (C/Al) current collector. Moreover, iron (Fe) dissolution and the deposition of dissolved Fe on the Li metal also contributed significantly to the degradation. Localized high‐concentration electrolytes (LHCEs) suppress iron dissolution and Li dendrite growth, resulting in long‐cycle stability for 300 cycles. This study provides insights into the further development of conversion‐type metal fluorides across various compositions.

## Graphical abstract

Porous micro‐sized FeF₂ is synthesized via a simple solid‐state process, forming a unique surface and internal structure to enhance capacity (570 mAh g^−1^). Further improvements in cycling stability are achieved through electrode modification to prevent delamination, and electrolyte engineering minimizes Fe dissolution, together ensuring long‐term performance and stability in FeF₂ batteries.

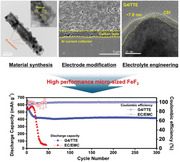

## 1 Introduction

Lithium‐ion batteries (LIBs) have attracted significant attention as environment‐friendly batteries for electric vehicles that align with eco‐friendly policies aimed at mitigating global warming and climate change and have been successfully used in various applications. However, there is a growing demand for LIBs with higher energy densities and lower costs for large‐scale applications such as electric transportation. Currently, LIBs employ intercalation‐based cathodes, such as lithium‐layered oxides (LiNi_1‐x‐y_Co_x_Mn_y_O_2_), which cannot satisfy these demands due to their limited capacities as they allow only one electron per transition metal to be active.^[^ ^1^ ^]^ Additionally, using nickel (Ni) and cobalt (Co) as transition metals is expensive and their prices fluctuate considerably, thereby failing to meet the demand for low‐cost LIBs.^[^ ^2^ ^]^ These limitations have underscored the need for developing new high‐energy‐density and low‐cost cathode materials. In contrast to intercalation‐based lithium‐layered oxides, iron (II) fluoride (FeF_2_) is considered a next‐generation cathode material. Owing to its high crustal abundance, Fe is significantly more cost‐effective than Co and Ni,^[^ ^3^ ^]^ making FeF_2_ more cost‐effective than other cathodes. Additionally, FeF_2_ has a high theoretical capacity of 571 mAh g^−1^ because it allows two electrons per transition metal to participate in the electrochemical reaction (FeF_2_ + 2Li^+^ + 2e^−^ → Fe + 2LiF).^[^ ^4^, ^5^ ^]^ Moreover, due to the high electronegativity of F, FeF_2_ exhibits a higher theoretical voltage (2.66 V vs Li/Li^+^) than other conversion‐reaction cathodes.^[^ ^4^, ^6^ ^]^ Furthermore, because of its high theoretical capacity and voltage, FeF_2_ exhibits a high energy density of up to 1519 Wh kg^−1^.^[^ ^7^ ^]^

Despite these advantages, numerous challenges must be addressed before FeF_2_ can be commercialized. First, its synthesis is challenging as impurity phases form easily because fluorine is volatile at high temperatures. Consequently, fluorine phases, including FeF_2_, are generally synthesized via solution‐based synthesis such as solvothermal or hydrothermal methods at low temperatures.^[^ ^8^ ^]^ However, these methods entail long synthesis times and result in low yields. Therefore, it is necessary to develop solid‐state synthesis methods for FeF_2_. Additionally, it has intrinsically poor ionic/electronic conducting characteristics,^[^ ^9^ ^]^ and its lithiated phase, LiF is insulating both ionically and electronically.^[^ ^10^ ^]^ Thus, many studies have focused on reducing the particle size of FeF_2_ to facilitate fast reactions by shortening the Li‐diffusion path length within the material.^[^ ^11^ ^]^ Additionally, the large surface area of nano‐FeF_2_ allows for better physical contact with conducting agents such as carbon, thereby enhancing electron conduction. A previous study showed that nano‐FeF_2_ integrated with a carbon matrix exhibits high reversible capacity.^[^ ^9^ ^]^ Conversely, micron‐sized metal fluorides demonstrate low reversible capacity even at high temperatures.^[^ ^12^ ^]^ Therefore, nano‐structuring is considered a prerequisite for their electrochemical reactions. However, nano‐structuring with non‐active materials (carbon) lowers the energy density.^[^ ^13^ ^]^ Additionally, the utilization of nanoparticles only lowers the packing density compared to the bimodal distribution.^[^ ^14^ ^]^ Thus, large FeF_2_ particles are required to improve the energy density of the electrode.

The third issue is poor cycle retention with large hysteresis and numerous Fe dissolutions,^[^ ^15^ ^]^ which reduces the active materials, leading to rapid capacity fading. Furthermore, FeF_2_ not only has poor conductivity but also undergoes significant volume change during (dis)charging.^[^ ^4^ ^]^ Owing to these poor material properties, achieving a reversible reaction in FeF_2_ over long cycles is considered challenging. However, stable cycle retention was recently achieved through electrolyte modification.^[^ ^16^ ^]^ For instance, by employing an ionic liquid (1 M lithium bis(fluorosulfonyl)imide (LiFSI)/Pyr1,3FSI), 90% of capacity can be maintained after 50 cycles due to the formation of a stable solid‐electrolyte interphase (SEI) layer on FeF_2_.^[^ ^11^ ^]^ Additionally, Qiao Huang et al. investigated various electrolytes and proposed using 3 M LiFSI in 1,2‐Dimethoxyethane (DME) to stabilize the electrochemical reaction.^[^ ^5^ ^]^ Thus, appropriate cathode‐electrolyte interphase (CEI) formation is crucial for long‐term reversible cycling. In addition to the cathode, the electrolyte is important for Li‐metal anodes employed in FeF_2_ full cells. Li metals tend to dendritic growth, which can cause short circuits and potentially lead to fires.^[^ ^17^ ^]^ Furthermore, Li dendrites contribute to the formation of dead Li, resulting in capacity fading and increased resistance.^[^ ^18^ ^]^ Therefore, electrolytes must be developed to ensure the stability of Li‐FeF_2_ batteries.

In this study, we comprehensively address and resolve these issues. First, we determined the theoretical capacity of micron‐sized FeF_2_ by synthesizing highly porous micron‐sized FeF_2_ via solid‐state synthesis. This novel method overcomes the limitations of nano‐structuring, which reduces the electrode energy density despite its high reactivity and contradicts the general knowledge that nano‐structuring is required to activate conversion‐type metal fluoride materials. Furthermore, we investigated the causes of capacity degradation and found that electrode delamination from the current collector due to the large volume change of FeF_2_ is the primary cause of capacity fading in the initial cycles. By replacing the Al current collector with a C/Al current collector, the adhesion between the electrode and the current collector was improved, thereby eliminating early‐cycle degradation. Additionally, in later cycles, using an appropriate electrolyte is crucial for stabilizing the Li‐FeF_2_ cell. Thus, this is the first study to propose an electrolyte formulation of 1.5 M  LiFSI in tetraethylene glycol dimethyl ether (G4)/1,1,2,2‐tetrafluoroethyl‐2,2,3,3‐tetrafluoropropylether (TTE) (3:7 (v/v)) for Li‐FeF_2_ batteries. This electrolyte suppresses Fe dissolution by forming an appropriate CEI layer on FeF_2_ and exhibits a stable reaction with Li metals. By improving the cathode material itself and optimizing the electrolyte and electrode high cyclability and capacity were achieved using micron‐sized FeF_2_. This effect is not limited to metal fluoride cells but shows great potential for application in other Li‐metal batteries as well. Thus, this study provides comprehensive insights into the utilization of conversion‐based cathode materials for next‐generation batteries.

## 2 Results

### 2.1 Synthesis of Micron‐Sized FeF_2_ and its Electrochemical Properties

**Figure 1:**
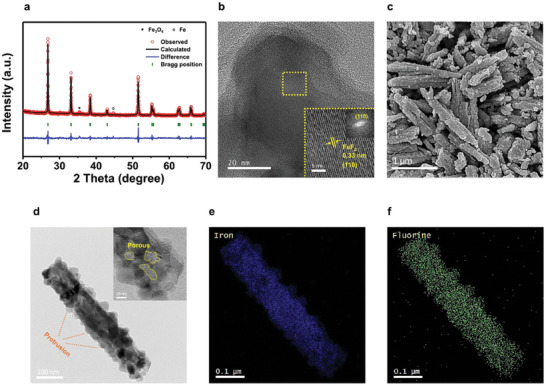
Characteristics of synthesized FeF_2_. a) Rietveld refinement profile of XRD data (R_p_: 7.32, R_exp_: 6.39, and Chi^2^: 2.19). b) HR‐TEM image of FeF_2_ particle and the inset is the enlarged view of the boxed area. c) SEM image of synthesized FeF_2_. d) TEM image of a single FeF_2_ particle. Inset presents an HR‐TEM image of the atomic layers. Elemental EDS mapping for e) Fe and f) F.

Metal fluorides (MF_X_, M = Fe, Cu, Mn, X = 2 or 3) are difficult to synthesize via solid‐state synthesis at high temperatures due to the high volatility of fluorine. As fluorine compounds can be synthesized using polytetrafluoroethylene (PTFE, (C_2_F_4_)_n_) as the fluorine precursor,^[^ ^19^ ^]^ we used it to synthesize FeF_2_ at 600 °C with a short annealing time of 1 h. The bulk structure analyzed via x‐ray diffraction (XRD) (**Figure** **1**a), exhibited a tetragonal structure (P4_2_/*mnm*) with lattice parameters of a = b = 4.70158(25), c = 3.30396(18), and α = ß = γ = 90°. Additional elemental analysis confirmed the composition of the synthesized FeF_2_. Fluorine content was measured using ion chromatography (IC), and iron was measured via inductively coupled plasma optical emission spectrometry (ICP‐OES) (Table S1, Supporting Information). The molar ratio of fluorine to iron was calculated as 2.07:1.05, which is very close to the ideal stoichiometric ratio of 2:1 for FeF_2_. This result indicates that FeF_2_ was successfully synthesized, as the elemental composition aligns well with the theoretical stoichiometry. Moreover, the d‐spacing of the (110) plane of FeF_2_ (0.332 nm) matched well, as confirmed by its fast Fourier transform (FFT) pattern obtained via high‐resolution transmission electron microscopy (HR‐TEM) (Figure 1b). The novelty of our synthesis lies in the particle shape and microstructure of the synthesized FeF_2_, which exhibited ≈1 µm‐long and 100 nm‐wide rod‐shaped particles with a uniform particle‐size distribution, as shown in Figure 1c. The particle surfaces are uneven, resembling the agglomeration of many small rod‐shaped particles. Additionally, the internal pores within the particles were observed through TEM, as shown in Figure 1d. This observation is further supported by the BJH analysis, which revealed an average pore size of 4–5 nm (Figure S1, Supporting Information), with a cumulative pore volume of 0.034 cm^3^ g^−1^, indicating a well‐developed porous structure (Table S2, Supporting Information). In addition, the particle shapes of iron oxide hydroxide (FeOOH), which was used as the Fe precursor (Figure S2, Supporting Information), indicate that the particle shape and size of FeF_2_ depend on the metal precursor, implying that the morphology of metal fluorides can be manipulated by controlling the metal precursor. In contrast to the precursor, a wider protrusion‐like structure was formed after crystallization into iron fluoride. As indicated in the energy‐dispersive X‐ray spectroscopy (EDS) shown in Figures 1e,f, the particles exhibited uniform distributions of Fe and F. The surface area of the synthesized FeF_2_ was 19.9 m^2^ g^−1^, which is relatively large given the 1 µm length of the rod particles. Commercial FeF_2_ has a considerably lower surface area (≈3.7 m^2^ g^−1^), even with smaller particles (≈500 nm), than the synthesized FeF_2_ shown in Figure S3 (Supporting Information). The unique protruding and internal porous structures result in a large surface area, despite the micron‐sized particles.

**Figure 2:**
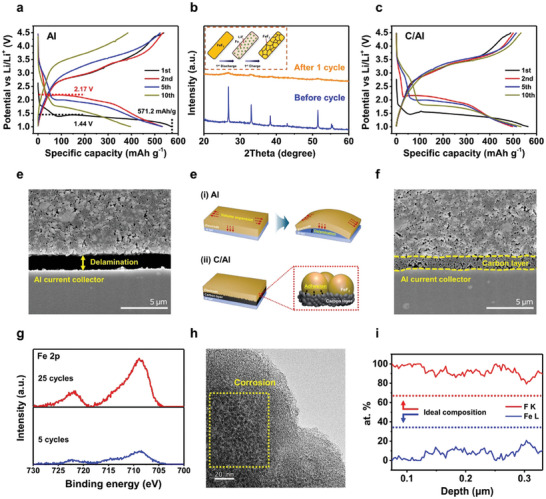
Electrochemical properties and degradation mechanisms of synthesized FeF_2_. Voltage profiles of Li‐FeF_2_ with a) Al, c) C/Al at 0.1C and 30 °C with 1.2 m LiPF_6_ in EC/EMC (3:7 (v/v)). b) XRD patterns of the electrode before and after 1 cycle. The inset schematic presents nano‐crystallization after the first cycle. Cross‐section images of the electrode after 5 cycles depending on the current collector, d) Al, f) C/Al. e) Schematic image presenting the different electrode behavior depending on the current collector. g) Fe 2P XPS results of Li‐metal anode after 5 and 25 cycles. h) TEM image of FeF_2_ particle after 25 cycles. i) Elemental atomic percentages of Fe and F by analyzing EELS line scanning.

The synthesized FeF_2_ exhibited a high discharge capacity of 571.2 mAh g^−1^ in the first cycle, which corresponded to its theoretical capacity (571 mAh g^−1^), as shown in **Figure** **2**a. This noteworthy achievement can be attributed to the unique microstructural characteristics of FeF_2_ that result in a high surface area. Although it exhibited a low discharge voltage of ≈1.44 V during the first discharge cycle, it increased to 2.17 V in the second cycle, as indicated in Figure 2a. This implies that (de)lithiation was considerably easier second cycle onward, indicating that a high activation energy is required for the first lithiation, and typical working voltages are obtained in subsequent cycles.^[^ ^7^, ^20^ ^]^

To determine the cause of this difference, the electrodes before and after the first cycle were analyzed through XRD, as shown in Figure 2b. Notably, the FeF_2_ peaks broadened significantly after the first cycle, indicating a reduction in crystallite size. However, this size reduction is not due to particle fracture. TEM images taken after 20 cycles confirm that the micron‐sized particles remain intact (Figure S4, Supporting Information). Therefore, while the overall particle size is maintained, nano‐crystallization occurs within the particles. This suggests that nano‐sized FeF_2_ grains form within the larger micron‐sized particles during charging after the first discharge cycle, allowing them to retain the characteristics of nanoparticles in subsequent reactions. These findings align with previous studies that observed the formation of small Fe crystals during the initial discharge state,^[^ ^11^, ^12^, ^21^ ^]^ and found that small crystals remained after lithiation without any significant growth in the crystal size, unlike in the initial state. These findings confirm that the behavior of FeF_2_ with large particles is similar to that of nanoparticles in subsequent reactions, demonstrating that micron‐sized metal fluorides can achieve a high capacity with low polarization due to nano‐crystallization. However, even though the theoretical capacity was achieved in the first cycle, the overpotential rapidly increased and the capacity decreased within 20 cycles, as shown in Figure S5 (Supporting Information).

### 2.2 Capacity‐Fading Mechanism of Li‐Micron‐Sized FeF_2_ Cell

The synthesized FeF_2_ with micron‐sized particles exhibited rapid capacity fading during the early cycles. The scanning electron microscopy (SEM) images of the electrode obtained after five cycles, as shown in Figure 2d, indicate that delamination occurred between the current collector and electrode layer. Consequently, the significant increase in the overpotential and capacity fading observed after five cycles was primarily due to the inability of the electron reaction from the current collector to the electrode layer. Delamination originates from substantial volume changes accompanying the FeF_2_ reaction. During charging and discharging, FeF_2_ undergoes a high volume expansion/contraction of ≈30%,^[^ ^15b^ ^]^ and as this process repeats over several cycles, the active material layer becomes completely delaminated from the current collector. The high volume change during the FeF_2_ reaction is an intrinsic characteristic of the material, which is attributed to its conversion‐reaction mechanism, unlike intercalation‐based materials.^[^ ^9^ ^]^ Therefore, instead of modifying the active material, we focused on inhibiting the delamination between it and the current collector. The Al current collector has a very smooth surface, which limits the contact area and results in weak adhesion.^[^ ^22^ ^]^ Therefore, we employed a C/Al current collector, which features a higher contact area with the active material, thereby enhancing adhesion, as shown in Figure 2e. No delamination was observed even after five cycles and a good contact between the current collector and active layer was evident, as shown in Figure 2f. To further investigate the effect of adhesion on electrochemical performance, electrochemical impedance spectroscopy (EIS) was conducted after five cycles. As shown in Figure S6 (Supporting Information), the charge transfer resistance of the C/Al was significantly lower compared to the Al, indicating improved interfacial contact and more efficient charge transfer. This result suggests that the C/Al not only enhances adhesion but also maintains a stable and uniform interface, preventing delamination and contributing to better electrochemical performance over cycling. Consequently, the overpotential during the early cycles (within 10 cycles) decreased, and a capacity of 535 mAh g^−1^ was maintained, as shown in Figure 2c.

However, the voltage hysteresis increased after 15 cycles and the capacity decreased over subsequent cycles, as shown in Figure S7 (Supporting Information). Therefore, post‐cycled electrodes were investigated to determine the cause of fading. Fe‐related peaks were detected in the Li‐metal even after the fifth cycle, and the intensities of the Fe peaks in the 25th cycle were significantly higher than those in the fifth cycle (Figure 2g). This indicated that Fe dissolution occurred in the initial cycles and progressed in subsequent cycling. Additionally. TEM observations after 25 cycles revealed corrosion and perforations in the particles (Figure 2h). Furthermore, the atomic percentages of Fe and F in the particles, obtained via electron energy loss spectroscopy (EELS), revealed that the Fe─F ratio deviated from the expected 1:2 (The corresponding EELS scanning pathway was shown in Figure S8, Supporting Information), indicating a significantly reduced proportion of Fe and confirming its substantial dissolution (Figure 2i). Thus, we found that delamination of the active layer and metal dissolution in the electrolyte were the main causes of capacity fading in the Li‐FeF_2_ cells.

### 2.3 Suppressed Fe Dissolution in Li‐FeF_2_ Cell by Localized High Concentration Electrolytes

As metal dissolution in FeF_2_ causes capacity degradation, an optimal electrolyte that can form a robust CEI to suppress this issue was required.^[^ ^23^ ^]^ LHCEs were considered a good option because they can effectively suppress active material dissolution and side reactions with Li‐metals by forming anion‐derived CEI and SEI layers due to abundant contact‐ion pairs (CIP) and cation–anion aggregates (AGG).^[^ ^24^ ^]^ In this study, G4 and DME were used as solvating solvents due to their good stability with Li‐metals,^[^ ^25^ ^]^ whereas TTE and 1,4‐Dioxane (DX) were used as the diluents.^[^ ^26^ ^]^ Moreover. LiFSI was used as the Li salt. In the density functional theory (DFT) calculations shown in Figure S9a (Supporting Information), the binding energies of Li‐ions with solvents were calculated in the order of G4, DME, DX, and TTE. Notably, G4 and DME exhibited higher binding energies than TTE and DX, suggesting that Li‐ions are more likely to bind to G4 and DME than to DX and TTE, indicating that they act as solvating solvents, whereas DX and TTE act as diluents. Three LHCEs were prepared with the same molar ratio of salt but using different solvents: 1.5 m LiFSI in G4/TTE, DME/TTE, and G4/DX. All solvents had a 3:7 vol% ratio of solvating solvent and diluent. The prepared electrolytes were observed using Raman spectroscopy, as shown in Figure S9b (Supporting Information). Three main peaks were observed for each electrolyte, corresponding to the solvating solvents (DME or G4), LiFSI bound to the solvating solvent, and TTE. Additionally, the formation of CIP or AGG was confirmed by the peak shift to a higher wavenumber in the 700–780 cm⁻¹ range, corresponding to the S─N─S stretching vibration of FSI⁻, in the 1.5 m electrolyte compared to the 0.5 and 1 M electrolytes as shown in Figures S9c,d (Supporting Information).^[^ ^27^ ^]^

**Figure 3:**
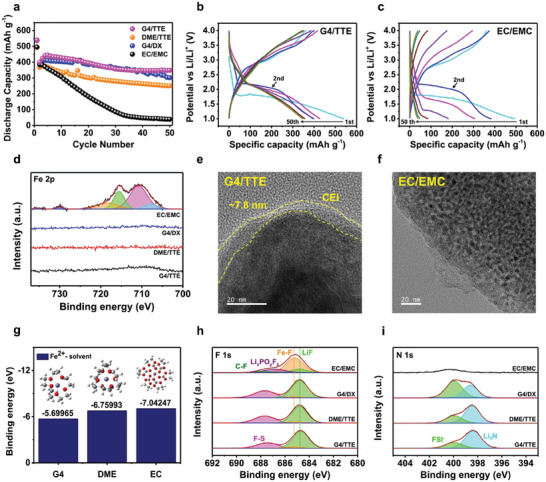
Electrochemical performance of Li‐FeF_2_ full cell with various electrolytes and their effect on FeF_2_. a) Capacity retention of Li‐FeF_2_ full cell tested at 0.1C and 30 °C. Voltage profiles of Li‐FeF_2_ full cell with b) G4/TTE, c) EC/EMC. d) Fe 2p XPS data of Li metal after 20 cycles. TEM images of FeF_2_ particles after 20 cycles with e) G4/TTE, f) EC/EMC. g) Binding energies of solvent with Fe^2+^ from DFT calculation and corresponding solvation structure (Violet: Fe, Red: O, Gray: C, White: H). XPS data of FeF_2_ after 20 cycles h) F 1s, i) N 1s.

Li‐FeF_2_ full cells were cycled using the prepared electrolytes and a reference (1.2 m lithium hexafluorophosphate (LiPF_6_) in ethylene carbonate (EC)/ethyl methyl carbonate (EMC) (3:7 (v/v)), as shown in **Figure** **3**a. Hereafter, all the electrolytes are named according to their solvent combinations: EC/EMC, G4/TTE, DME/TTE, and G4/DX. To exclude the influence of the specific morphology of the synthesized FeF_2_ and assess the effects of the electrolytes, commercially available FeF_2_ was used as the active material. All the cells exhibited high first‐discharge capacities: G4/TTE, 540 mAh g^−1^; DME/TTE, 492 mAh g^−1^; G4/DX, 538 mAh g^−1^; and EC/EMC, 495 mAh g^−1^, which closely approximated the theoretical capacity of FeF_2_ (571 mAh g^−1^). When LHCEs were used, significantly improved capacity retention was observed compared to EC/EMC. With EC/EMC, significant capacity loss occurred after the 30th cycle, whereas this was greatly mitigated with LHCEs. Additionally, when the second discharge capacity was considered as the initial capacity owing to the activation reaction occurring during the first discharge cycle, only 10% of the capacity was retained by the 50th cycle in EC/EMC. In contrast, LHCEs, specifically G4/TTE, DME/TTE, and G4/DX exhibited stable capacity retentions of 87, 68.2, and 76.8%, respectively, at the 50th cycle, indicating that the LHCEs effectively enhanced the cycling stability of the Li‐FeF_2_ full cells. Relative to that of G4/TTE, the capacities of the other LHCEs decreased more rapidly with cycling. However, the capacity fading was significantly less severe than that with the EC/EMC. The voltage profiles of G4/TTE and EC/EMC are presented in Figures 3b,c, wherein it is evident that large polarization occurs during cycling in the EC/EMC, resulting in rapid capacity fading. Severe voltage decay, as seen with EC/EMC, did not occur with G4/TTE, and from the 30th cycle onward, the working voltages became saturated. The voltage profiles obtained using other LHCEs are shown in Figure S10 (Supporting Information). Additionally, the differential capacity versus voltage (dQ/dV) profiles are presented in Figure S11 (Supporting Information), which shows the overpotential tendency more clearly. In the EC/EMC, the working voltage decreased from 2.12 V in the second cycle to 1.27 V in the 20th cycle. In contrast, the working voltages with G4/TTE, DME/TTE, and G4/DX decreased from 2.12 V in the second cycle to 2.08, 2.09, and 2.03 V, respectively, in the 20th cycle. Additionally, distinct differences were observed in the working voltages during the charged states. A rapid decrease in intensity with cycling occurred in the EC/EMC, and the charge reaction was nearly imperceptible around the 20th cycle. In contrast, the charging reaction was observable even after the 20th cycle in the LHCEs, indicating the high reversibility of the electrochemical reactions.

### 2.4 Improved Li Metal Stability by Localized High Concentration Electrolytes

To determine the cause of the improved cell performance observed in the LHCEs, the post‐cycled electrodes were analyzed after 20 cycles. In FeF_2_, Fe dissolution has been identified as the primary cause of degradation, so it was investigated first. As shown in Figure 3d, a high‐intensity peak was observed in the Fe 2p spectrum of the Li‐metal with EC/EMC, offering direct evidence of Fe dissolution during cycling and Fe deposition on the Li‐metal. Conversely, negligible peaks were detected in the Fe 2p region when LHCEs were used, indicating negligible Fe dissolution and demonstrating that LHCEs can effectively suppress Fe dissolution. DFT calculations were conducted to compare the solvating preference of Fe^2+^, as shown in Figure 3g. Considering the binding energies of Fe^2+^ and the solvent, EC exhibited the strongest binding to Fe^2+^, whereas G4 and DME showed weaker binding energies. This implies that EC has a higher propensity to bind with Fe, potentially leading to increased Fe dissolution in EC/EMC. Moreover, iron dissolution was less likely to occur in the LHCEs.

The cycled cathode and FeF_2_ particles were observed using TEM, as shown in Figure 3e, wherein an ≈7.8 nm‐thick uniform CEI layer can be observed on the particles when the G4/TTE electrolyte was used. This layer helps suppress Fe dissolution during cycling.^[^ ^28^ ^]^ In contrast, CEI in the EC/EMC (Figure 3f) is either almost absent or primarily present in localized regions. As a result, reactions with the electrolyte likely occurred, leading to iron dissolution. Moreover, the particles appeared to be corrosive owing to metal dissolution. The CEI compositions were investigated using X‐ray photoelectron spectroscopy (XPS), as shown in Figures 3h,i. LHCEs exhibited high intensities of FSI^−^ and Li_3_N at 399.9 and 398.4 eV,^[^ ^29^ ^]^ respectively, and LiF and F‐S bindings at 648.8 and 687.5 eV, respectively,^[^ ^30^ ^]^ indicating the formation of anion‐derived CEIs by LHCEs. In particular, because LiF and Li_3_N are known to efficiently suppress active material dissolution, the CEI composition helps improve cycling stability.^[^ ^28^, ^31^ ^]^ By contrast, the CEI layer in EC/EMC does not have an N‐based layer and instead forms a solvent‐derived CEI that is more organic‐rich than LHCEs (Figure S12a, Supporting Information). Furthermore, besides the weak LiF peak, an additional Fe─F bonding peak at ≈685 eV in F 1s was observed in the EC/EMC, indicating that some FeF_2_ was exposed to the electrolyte as all particles were not covered with the CEI layer. This was consistent with the TEM results. Owing to the non‐uniform CEI, the exposed metal fluoride deteriorates the stability of the electrochemical reaction.

**Figure 4:**
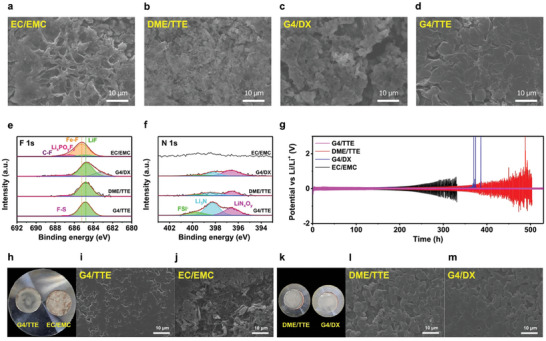
Effects of electrolytes on Li metal in Li‐FeF_2_ full cell and Li/Cu cells. Morphology of Li metal in a) EC/EMC, b) DME/TTE, c) G4/DX, d) G4/TTE in Li‐FeF_2_ full cell. The cells were disassembled after 20 cycles, except for G4/DX which was analyzed after 50 cycles. Investigation of the surface of Li metal by XPS e) F 1s, f) N 1s after 20 cycles. g) Voltage profiles of Li/Li symmetric cell at 1 mAh cm^−2^. h,k) Digital images Cu foil after Li deposition. Micro‐scale morphology analysis of deposited Li on Cu foil by SEM i) G4/TTE, j) EC/EMC, l) DME/TTE, and m) G4/DX.

Even though Fe dissolution was determined to be the primary cause of the performance degradation in the Li‐FeF_2_ cell. However, with prolonged cycling, the influence of side reactions with Li, such as dendrite formation and dead Li, gradually increases, leading to significant performance degradation of Li‐FeF_2_ full cells. Therefore, achieving stability with Li‐metals is important for obtaining a highly stable full cell. As LHCEs are known to stabilize Li‐metals, they are expected to positively affect their stability.^[^ ^32^ ^]^ Morphologies of the Li deposited in each electrolyte after the 20th cycle were observed by SEM, as shown in **Figures** **4**a–d. Needle‐like Li dendrite growths were observed in the EC/EMC electrolyte, whereas vertical dendrite growths were not observed in DME/TTE. However, Li was deposited unevenly. In G4/DX, the same non‐uniform Li deposition was also confirmed. In G4/TTE, Li deposited as flat and uniformly large granules, with a nearly constant height and no dendrites. This indicates that G4/TTE had the highest anodic stability.

Although LHCEs have the same salt molar ratio, they can have different HOMO/LUMO levels owing to their different solvents and molecular structures. This difference can lead to the formation of SEI layers with different compositions of Li metals.^[^ ^33^ ^]^ The SEI composition of Li in various electrolytes was investigated by XPS, as shown in Figures 4e,f. In the EC/EMC, a large peak corresponding to Fe─F was observed owing to Fe dissolution, and no N‐layer was observed. By contrast, all the SEI layers formed by LHCEs were composed of LiF‐rich, LiN_x_O_y_, and Li_3_N compounds. LiF enables uniform Li deposition by lowering the energy barrier compared to other inorganic SEI components and features a higher mechanical strength than Li‐metal (Young's modulus of 64.9 vs 7.82 GPa), which contributes to the flat Li deposition.^[^ ^34^ ^]^ Moreover, Li_3_N can promote fast Li^+^ deposition owing to its exceptionally high Li‐ionic conductivity (≈10^−3 ^S cm^−1^), effectively inhibiting dendrite growth.^[^ ^35^ ^]^ Thus, LiF and Li_3_N‐rich SEI were observed in only G4/TTE as fast Li‐ion conduction on the SEI layer is crucial for depositing flat and large granules on Li‐metals.

**Figure 5:**
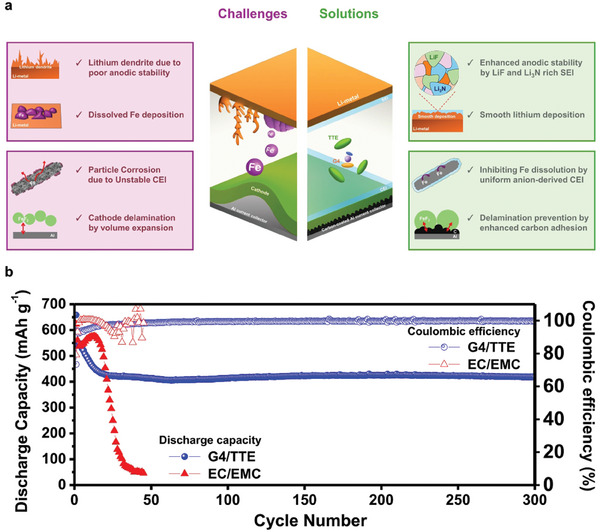
Cycle stability of micron‐sized FeF_2_ with applying our solutions. a) Schematic image representing challenges and solutions of the conversion‐type FeF_2_. b) Discharge capacity retention and coulombic efficiencies of Li‐synthesized FeF_2_ full cell with G4/TTE and EC/EMC tested at 0.1C.

The dependence of the stability of the Li‐symmetric cell on the electrolytes is shown in Figure 4g. In the EC/EMC, the polarization began to increase rapidly, and a large voltage shake occurred after ≈300 h, whereas DME/TTE exhibited good performance for up to 320 h. However, beyond this point, the overpotential increased rapidly, and from ≈380 h, a significant voltage shift was observed. Although the degradation in DME/TTE was delayed compared with EC/EMC, the overall trend was similar. G4/DX exhibited a significantly low overpotential and was stable for up to 350 h. However, after 370 h, a rapid increase in polarization occurred, resulting in sharp voltage spikes over 2.5 V. Subsequently, the reaction voltage almost converged to zero, indicating an internal short‐circuit, which can lead to thermal runaway owing to the excessive current and potentially cause cell ignition. In contrast, the G4/TTE cell exhibited remarkable stability and reversible reactions, with no significant increase in overpotential even after 500 h. Thus, G4/TTE offers the most stable Li‐metal reaction among all electrolytes. Additionally, a Li/Cu cell was tested using these electrolytes by depositing Li ions onto the Cu foil. Digital images of the Li deposited on Cu are shown in Figures 5h,k. In EC/EMC, non‐uniform Li deposition was observed, even with the naked eye. In contrast, no areas without lithium deposition were observed on the Cu foil with LHCEs, except for a small region at the end of the foil. This is owing to the formation of stable SEI, such as LiF and Li_3_N, in the LHCEs, leading to facile Li deposition and diffusion. The difference was even more distinct in micro‐scale observations of Li deposition via SEM. A non‐uniform Li deposition was confirmed in the Cu foil of the EC/EMC (Figure 4j), even at the micro‐scale, and particles with a thin and needle‐like dendritic morphology were observed. In contrast, Li depositions in the LHCEs exhibited more uniform and larger granules. Among these, G4/TTE (Figure 4i) exhibited the most uniform and flat deposition morphology. Thus, 1.5 M LiFSI in G4/TTE can be considered a suitable electrolyte for Li‐metal batteries.

### 2.5 Long‐Term Stability of the Li‐Micron‐Sized FeF_2_ Full Cell

**Figure** **5**a summarizes the challenges of the Li‐FeF_2_ full cell and their solutions. From our material development, micron‐sized FeF_2_ achieved high capacity due to the unique microstructure. However, severe capacity fading appears. To improve the stability, the capacity fading mechanism was revealed and resolved. C/Al current collector successfully inhibits electrode delamination by enhanced adhesion by carbon. Additionally, Fe dissolution and deposition on Li metal were observed in the 1.2 M LiPF_6_ in EC/EMC (3:7, (v/v)). Fe dissolution can be suppressed by the LHCEs by providing a stable CEI layer. Among them, 1.5 M LiFSI in G4/TTE (3:7, (v/v)) exhibits the most stable capacity retention with surprisingly high stability with Li‐metal by forming stable SEI layers. Using the optimal electrolytes, a full cell with the micron‐sized FeF_2_ was tested at 0.1C in Figure 5b. The greatly improved capacity retention of 78% has been maintained after 300 cycles, with a coulombic efficiency of > 99% in G4/TTE compared to EC/EMC. In Figure S13 (Supporting Information), the performance of the micron‐sized FeF₂ is compared with other FeF₂ materials, highlighting its notable stability even at such a large particle size. Despite the large volume changes typically seen in conversion‐type particles, this cell exhibits highly stable performance. The electrolyte effectively stabilizes both the metal fluoride and Li metal by forming CEI and SEI layers (Figure 5a). Its voltage profile is shown in Figure S14a (Supporting Information). Polarization and capacity fading during cycling were suppressed. Furthermore, even with a carbon ratio of 70% and an active material loading of 1.6 mg cm^−^^2^, which is comparable to the loading levels reported in other metal fluoride studies,^[^ ^7^, ^11^, ^15^, ^36^ ^]^ the cell demonstrated excellent cycle stability, maintaining a capacity of 376 mAh g^−1^ after the 70th cycle, as shown in Figure S15 (Supporting Information). This performance is comparable to other studies on nano‐sized FeF₂, despite the fact that the FeF₂ particles used in this study are micron‐sized. Achieving such high capacity and cycle stability with a carbon ratio of 70% highlights the strength of our work, especially given the challenges typically associated with using larger particles. This excellent performance is attributed to the uniform and effective formation of the CEI across the entire surface of the synthesized FeF₂ particles. EDS measurements in Figure S16 (Supporting Information) revealed that nitrogen (N), oxygen (O), and sulfur (S) were consistently detected throughout the particle, confirming that the CEI was uniformly and successfully formed. This uniform CEI formation played a crucial role in enhancing cycling stability by preventing Fe dissolution during cycling. The rate capability of the micron‐sized FeF_2_ using the optimal electrolyte was tested with the same charge and discharge rates up to a C‐rate of 2C, exhibiting capacities of 342, 195, and 141 mAh g^−1^ at 0.2, 0.5, and 1C, respectively (Figure S14b, Supporting Information). The rate performance appears to be reasonable considering the particle size and conversion mechanism. These results demonstrate the feasibility of micron‐sized FeF_2_ for achieving high energy density.

## 3 Discussion

### 3.1 Utilization of Micron‐Sized Iron Fluoride

Because a bimodal particle distribution is commercially utilized to increase the energy density of electrodes, both nano and micron‐sized particles of FeF_2_ must be investigated. However, because of their poor conducting properties, previous studies have predominantly focused on nano‐sized materials. This paper presents novel and significant advances in the development and utilization of FeF_2_ with large particles. Unlike the low capacity typically obtained with large metal fluoride particles, the FeF_2_ synthesized in this study achieved its theoretical capacity. Notably, nano‐crystallization was observed after the first discharge reaction, resulting in reduced polarization in subsequent cycles. This indicates that with appropriate microstructural modifications, the initial activation reaction of microparticles can be overcome, making them sufficiently usable. Additionally, the electrochemical performance of micron‐sized FeF_2_ can be further enhanced by material modification such as the introduction of various conducting agents, similar to those used with nano‐sized FeF_2_.^[^ ^15^, ^21^ ^]^ This, along with the potential for increasing particle size, holds promise for significant improvements. However, it is important to note that micron‐sized particles have a lower surface‐area‐to‐volume ratio compared to nanoparticles. This characteristic hinders effectively releasing the strain‐stress energy associated with the (dis)charge reaction to the surface, which can lead to electrode delamination from the current collector during (dis)charging. Therefore, to utilize micron‐sized conversion‐type materials with large volume changes, it is important to ensure strong adhesion between the electrode and current collector or to form a matrix that can accommodate the volume changes.

### 3.2 Superiority of the Proposed Electrolyte in Li‐FeF_2_ Batteries

During (dis)charging of the Li‐FeF_2_ batteries, iron dissolution was observed owing to the lack of a CEI layer on the FeF_2_. Previous studies have shown that protective conducting layers on FeF_2_, such as carbon coatings, polymer complexes, and embeddings, can suppress material dissolution and stabilize the reaction.^[^ ^7^, ^9^, ^15^ ^]^ However, the simplest way to form a protective layer on FeF_2_ is to establish a stable CEI layer during the electrochemical reaction, which critically depends on the electrolyte used. In this study, 1.5 m MLiFSI in G4/TTE (3:7 (v/v)) was demonstrated to be the most superior electrolyte for Li‐FeF_2_ batteries. First, G4/TTE successfully suppressed Fe dissolution by the uniform anion‐derived CEI and exhibited a lower reactivity with Fe^2+^ than EC and DME. More importantly, because of the long molecular structure of G4, it surrounds and binds with Li‐ions, resulting in a stronger binding energy than DME (Figure S9a, Supporting Information), even when only one G4 solvent coordinates with one Li‐ion (Figures S9e,f, Supporting Information). Consequently, G4‐based electrolytes result in weaker binding between FSI^−^ and Li^+^ compared to DME‐based electrolytes, as evidenced by the Raman spectra peak of G4/TTE appearing at a lower wavenumber than that of DME/TTE in Figures S9c,d (Supporting Information). Additionally, because TTE has a low binding energy with Li‐ions, it can form LHCE solvation structures more effectively. However, because DX has a relatively high binding energy, some Li ions may bind to it, potentially disrupting the solvation‐structure formation of LHCE. Thus, G4/TTE can easily form anion‐derived SEI, and most of the Li_3_N‐rich SEI layers in G4/TTE were observed after cycling. This finding indicates that the binding energy of the solvent with the salt should be considered when selecting solvents for (L)HCEs. Furthermore, it is expected to contribute to the development of other Li metal–conversion cathode batteries as well.

## 4 Conclusion

We successfully synthesized micron‐sized FeF_2_ with a unique protrusion surface and internal pore via a simple solid‐state synthesis method. Owing to its unique microstructure and large surface area, a high capacity of 571 mAh g^−1^ was achieved even with large particle sizes. However, rapid capacity fading was observed, which was attributed to the delamination between the current collector and electrode because of the large volume changes during the FeF_2_ reactions. This issue was addressed by replacing the conventional Al current collector with a C/Al current collector to prevent delamination. Nevertheless, serious capacity degradation was still observed in the subsequent cycles, which was attributed to iron dissolution, and various LHCEs were used to suppress it. All cells using the LHCEs exhibited improved cycle stability compared to that using 1.2 M LiPF_6_ in EC/EMC (3:7 (v/v)). The LHCEs effectively prevented iron dissolution by forming anion‐derived CEI layers. Furthermore, the ether‐based LHCEs demonstrated higher stability with Li‐metals than carbonate electrolytes. Among the LHCEs, G4/TTE exhibited the most stable cycle retention in both the full and Li‐symmetric cells. Notably, the morphology of the Li deposition in G4/TTE exhibited flat and large granules due to the Li_3_N/LiF‐rich SEI layer. By using this electrolyte, Li‐cells with micron‐sized FeF_2_ achieved long‐cycle stability for 300 cycles at 0.1C. Thus, this study demonstrated the feasibility of using large‐particle conversion‐type metal fluorides with appropriate electrolytes.

## 5 Experimental Section

### Material Synthesis and Electrode Manufacturing

FeF_2_ was synthesized via a solid‐state reaction using FeOOH and PTFE (Sigma–Aldrich) as the starting materials. These were mixed in a 1:1.2 mass ratio in a mortar until a homogeneous mixture was achieved. The mixture was pelletized and then calcined at 600 °C for 1 h under an argon atmosphere in a tube furnace, resulting in the final FeF_2_ product. For electrode fabrication, a slurry was prepared by mixing the synthesized FeF_2_, acetylene black, and polyvinylidene fluoride (PVDF) in a mass ratio of 70:25:5. This slurry was applied to both Al and C/Al current collectors using a Baker‐type applicator. When using a carbonate electrolyte, the active material loading for the electrodes with synthesized FeF_2_ was 1.95 mg cm^−^^2^ on the C/Al current collector (30 µm thickness) and 1 mg cm^−^^2^ on the bare Al current collector.

For the application of the G4/TTE electrolyte to the synthesized FeF_2_, electrodes were fabricated with two different mass ratios: a 50:45:5 (active material: acetylene black: PVDF) ratio, where the active material loading was 0.5–0.6 mg cm^−^^2^, and a 70:25:5 ratio, where the active material loading was 1.6 mg cm^−^^2^. Both electrodes used a C/Al current collector with a thickness of 30 µm. To investigate the performance of LHCEs on commercial FeF_2_ (Sigma‐Aldrich), electrodes with a mass ratio of 70:25:5 were prepared, and the active material loading was between 1.0‐1.2 mg cm^−^^2^. These are clarified in supporting information (Table S3, Supporting Information).

### Electrolyte Materials and Preparation

LiFSI (>98%), DME (>99%), TTE(>95%), and DX (>99%) were purchased from TGI·SEJIN CI. G4 (98%) was obtained from Alfa Aesar. All electrolytes were prepared in an Ar‐filled glovebox with a 0‐ppm concentration of H_2_O and <0.3‐ppm concentration of O_2_. First, 0.28,059 g (1.5 mmol) of LiFSI was dissolved in 0.3 mL of solvating solvent (G4, DME) and allowed to stand for 3 days until LiFSI was completely dissociated. This is a 5 m high‐salt concentration electrolyte (HCE) wherein LiFSI bonds with only a solvating solvent. Subsequently, 0.7 mL of diluent (TTE, DX) was added to the HCE and the mixture was stirred to obtain an LHCE with 1.5 M LiFSI in the solvating solvent/diluent (3:7 (v/v)). The carbonate electrolyte of 1.2 m LiPF_6_ in EC/EMC (3:7 (v/v)) was purchased from Dongwha Electrolyte.

### Full‐cell, Li/Li‐Cell, Li/Cu‐Cell Construction

Lithium metal with a thickness of 200 µm was punched into circular discs with a diameter of 15 mm, while the cathode was punched into 13 mm circles. Each cell type was assembled using a CR2032 coin cell. For full‐cell construction, the assembly included a 13 mm circular cathode, 120 µL of electrolyte, a polypropylene (PP) monolayer separator (Celgard 2400), and a 15 mm circular lithium metal anode.

For Li/Li‐Cell construction, two lithium metal discs (200 µm thickness, 15 mm diameter) were used as both the working and counter electrodes.

For Li/Cu‐Cell construction, 20 µm thick copper foil was treated with 1 M hydrochloric acid (HCl) in deionized water to remove the native oxide layer, followed by thorough washing with deionized water. The treated Cu foil was then punched into 15 mm discs and used as the working electrode.

### Physical Characterization

The crystal structures of the synthesized FeF_2_ and the electrodes were analyzed using XRD on a Bruker D8 ADVANCE instrument, employing Cu Kα radiation (λ = 1.54060 Å). The measurements were performed in the 2θ ranges of 20–70° for the synthesized FeF_2_ and 20–60° for the electrodes. The surface area of the samples was determined through Brunauer–Emmett–Teller analysis using a BELSORP‐mini II, conducted under a nitrogen atmosphere at a pressure range of 0–1 bar and a temperature of 77 K.

The morphological analysis of the samples was performed via SEM using a JSM‐7401F (JEOL LTD, Japan). For detailed cross‐sectional imaging of the electrodes, samples were prepared using an ion milling system equipped with an ArBlade 5000 (Hitachi High‐Tech), and then further examined using field emission scanning electron microscopy with the Inspect F instrument.

The elemental analysis was conducted using various instruments: Fe content was measured using a 5800 ICP‐OES (Agilent), F content was determined using a 930 Compact IC Flex (Metrohm), and C content was analyzed using an ELEMENTRAC CS‐d (Eltra).

XPS measurements were conducted using a monochromatic Al Kα source (λ = 1486.6 eV) under ultra‐high vacuum conditions (<5.0 × 10^‐9 mbar). All XPS spectra were calibrated using the carbon─carbon bond peak at 285 eV as a reference. TEM images were obtained using an FEI Titan 80–300 to further analyze the microstructure of the samples.

### Electrochemical Characterization

All electrochemical tests were conducted at 30 °C using the NEWARE BTS system. For the galvanostatic tests of the full cells, carbonate electrolyte cells were cycled within voltage windows of 1–4 V and 1–4.5 V (vs Li/Li^+^), while cells using LHCEs were tested within a voltage window of 1–4 V (vs Li/Li^+^). The narrower voltage window for LHCEs was due to their lower oxidative stability compared to carbonate‐based electrolytes.

The full cells were cycled at a rate of 0.1C (corresponding to 57.1 mA g⁻¹). In the Li/Li symmetric cell tests, lithium was stripped and plated for 1 h at a current density of 1 mAh cm⁻^2^. For the Li/Cu cell tests, lithium was deposited onto the Cu electrode at a current density of 1 mAh cm⁻^2^ for 2 h.

To evaluate the rate capability of the full cell with the G4/TTE electrolyte and the synthesized FeF_2_ cathode, the cells were cycled for 10 consecutive cycles at increasing C‐rates of 0.1, 0.2, 0.5, 1, and 2C. Afterward, the cells were returned to the initial rate of 0.1C for additional cycling. An electrochemical impedance spectroscopy (EIS) test was conducted using a Biologic electrochemical potentiostat/galvanostat device in a frequency range from 10 to 1 MHz.

### DFT Calculations

All DFT calculations were performed using the Gaussian 16 software package. The geometries of all solvents and their complexes with Li^+^ were optimized using the B3LYP density functional method at the 6–311+G(d,p) level. At the same level of theory, binding energies were calculated for the complexes in their ground states. Solvation effects were considered using the Polarizable Continuum Model (PCM) with THF as the explicit solvent (Eps = 7.4257, Eps(inf) = 1.9881).

## Conflict of Interest

The authors declare no conflict of interest.

## Author Contributions

C.C. and H.Y. equally contributed to this work. C.C., H.Y. and M.K. conceived the original idea, conducted experiments, and wrote the manuscript. S.K. and D.‐J.Y. performed DFT calculations and discussed the results. D.‐i.K. and J.H. performed an SEM analysis and discussed the results. M.S. performed Raman spectroscopy and discussed the results.

## Supporting information



Supporting Information

## Data Availability

The data that support the findings of this study are available from the corresponding author upon reasonable request.

## References

[1] M. S. Whittingham , C. Siu , J. Ding , Acc. Chem. Res. 2018, 51, 258. 29327579 10.1021/acs.accounts.7b00527

[2] a) X.‐Q. Wang , T. Wu , H. Zhong , C.‐W. Su , Resour. Policy 2023, 83, 103707;

[3] a) E. Gerold , R. Lerchbammer , H. Antrekowitsch , Metals 2022, 12, 1706;

[4] H. Song , H. Cui , C. Wang , J. Mater. Chem. A 2015, 3, 22377.

[5] Q. Huang , K. Turcheniuk , X. Ren , A. Magasinski , D. Gordon , N. Bensalah , G. Yushin , Adv. Energy Mater. 2019, 9, 1803323.

[6] L. Sun , Y. Li , W. Feng , Small Methods 2023, 7, e2201152. 36564355 10.1002/smtd.202201152

[7] a) B. R. Wygant , N. B. Schorr , I. V. Kolesnichenko , T. N. Lambert , ACS Appl. Energy Mater. 2022, 5, 13346;

[8] a) X. Zhang , Q. Zeng , J. Ji , H. Li , X. Ma , W. Fei , X. Guo , X. Zhou , Combust. Flame 2024, 261, 113298;

[9] W. Gu , A. Magasinski , B. Zdyrko , G. Yushin , Adv. Energy Mater. 2015, 5, 1401148.

[10] J. Pan , Y.‐T. Cheng , Y. Qi , Phys. Rev. B 2015, 91, 134116.

[11] A. W. Xiao , H. J. Lee , I. Capone , A. Robertson , T. U. Wi , J. Fawdon , S. Wheeler , H. W. Lee , N. Grobert , M. Pasta , Nat. Mater. 2020, 19, 644. 32094491 10.1038/s41563-020-0621-z

[12] K. Karki , L. Wu , Y. Ma , M. J. Armstrong , J. D. Holmes , S. H. Garofalini , Y. Zhu , E. A. Stach , F. Wang , J. Am. Chem. Soc. 2018, 140, 17915. 30456949 10.1021/jacs.8b07740

[13] a) Y. Wang , H. Li , P. He , E. Hosono , H. Zhou , Nanoscale 2010, 2, 1294; 20820717 10.1039/c0nr00068j

[14] C.‐H. Lin , S.‐K. Parthasarathi , S. Bolloju , M. Abdollahifar , Y.‐T. Weng , N.‐L. Wu , Energies 2022, 15, 8129.

[15] a) Q. Huang , T. P. Pollard , X. Ren , D. Kim , A. Magasinski , O. Borodin , G. Yushin , Small 2019, 15, e1804670; 30645034 10.1002/smll.201804670

[16] a) Y. Yu , C. Lai , M. Lei , K. Chen , C. Li , Mater Horiz 2024, 11, 2169; 38384254 10.1039/d3mh01908j

[17] S. Wang , K. Rafiz , J. Liu , Y. Jin , J. Y. S. Lin , Sustain. Energy Fuels 2020, 4, 2342.

[18] R. Zhang , X. Shen , Y.‐T. Zhang , X.‐L. Zhong , H.‐T. Ju , T.‐X. Huang , X. Chen , J.‐D. Zhang , J.‐Q. Huang , J. Energy Chem. 2022, 71, 29.

[19] M. Kim , S. Lee , B. Kang , Adv. Sci. 2016, 3, 1500366. 10.1002/advs.201500366 PMC5064735 27774395

[20] a) A. Kitajou , H. Yamagishi , M. Katayama , K. Yoshii , M. Shikano , H. Sakaebe , S. Okada , J. Electroanal. Chem. 2022, 920, 116577;

[21] a) Y. Su , J. Chen , H. Li , H. Sun , T. Yang , Q. Liu , S. Ichikawa , X. Zhang , D. Zhu , J. Zhao , L. Geng , B. Guo , C. Du , Q. Dai , Z. Wang , X. Li , H. Ye , Y. Guo , Y. Li , J. Yao , J. Yan , Y. Luo , H. Qiu , Y. Tang , L. Zhang , Q. Huang , J. Huang , Adv. Sci. 2022, 9, e2201419; 10.1002/advs.202201419 PMC9313485 35567353

[22] E.‐C. Cho , C.‐W. Chang‐Jian , Y.‐J. Wu , S.‐H. Chao , J.‐H. Huang , K.‐C. Lee , H. C. Weng , S.‐C. Hsu , J. Power Sources 2021, 506, 230060.

[23] K. Chen , M. Lei , Z. Yao , Y. Zheng , J. Hu , C. Lai , C. Li , Sci. Adv. 2021, 7, eabj1491. 34730994 10.1126/sciadv.abj1491 PMC8565847

[24] a) X. Ren , P. Gao , L. Zou , S. Jiao , X. Cao , X. Zhang , H. Jia , M. H. Engelhard , B. E. Matthews , H. Wu , H. Lee , C. Niu , C. Wang , B. W. Arey , J. Xiao , J. Liu , J. G. Zhang , W. Xu , Proc. Natl. Acad. Sci. 2020, 117, 28603; 33144505 10.1073/pnas.2010852117 PMC7682554

[25] X. Ren , L. Zou , S. Jiao , D. Mei , M. H. Engelhard , Q. Li , H. Lee , C. Niu , B. D. Adams , C. Wang , J. Liu , J.‐G. Zhang , W. Xu , ACS Energy Lett. 2019, 4, 896.

[26] a) J. Liu , B. Yuan , L. Dong , S. Zhong , Y. Ji , Y. Liu , J. Han , C. Yang , W. He , Batter. Supercaps 2022, 5, e202200256;

[27] T. D. Pham , A. Bin Faheem , K. K. Lee , Small 2021, 17, e2103375. 34636172 10.1002/smll.202103375

[28] W. Liu , J. Li , W. Li , H. Xu , C. Zhang , X. Qiu , Nat. Commun. 2020, 11, 3629. 32686673 10.1038/s41467-020-17396-x PMC7371675

[29] a) H. Li , L. Li , J. Zheng , H. Huang , H. Zhang , B. An , X. Geng , C. Sun , ChemSusChem 2023, 16, 202202220; 10.1002/cssc.202202220 36892939

[30] a) Y. Peng , K. Nishikawa , K. Kanamura , J. Electrochem. Soc. 2022, 169, 060548;

[31] a) Q. Zhang , J. Ma , L. Mei , J. Liu , Z. Li , J. Li , Z. Zeng , Matter 2022, 5, 1235;

[32] T. Li , Y. Li , Y. Sun , Z. Qian , R. Wang , ACS Mater. Lett. 2021, 3, 838.

[33] Y. He , Y. Zhang , P. Yu , F. Ding , X. Li , Z. Wang , Z. Lv , X. Wang , Z. Liu , X. Huang , J. Energy Chem. 2020, 45, 1.

[34] a) J. Zheng , Z. Ju , B. Zhang , J. Nai , T. Liu , Y. Liu , Q. Xie , W. Zhang , Y. Wang , X. Tao , J. Mater. Chem. A 2021, 9, 10251;

[35] a) S. Ni , M. Zhang , C. Li , R. Gao , J. Sheng , X. Wu , G. Zhou , Adv. Mater. 2023, 35, 2209028; 10.1002/adma.202209028 36482265

[36] Y. Xu , W. Xiong , J. Huang , X. Tang , H. Wang , W. Liu , D. Xiao , Y. Guo , Y. Zhang , J. Energy Chem. 2023, 79, 291.

