# Supplementary material for: Achieving High Stability and Capacity in Micron‐Sized Conversion‐Type Iron Fluoride Li‐Metal Batteries
Source: Adv Sci (Weinh). 2024 Oct 23;11(46):2410114. doi: 10.1002/advs.202410114 (PMC11633498; doi:10.1002/advs.202410114)
Supplement: Supplementary file 1 — Supporting Information [file ADVS-11-2410114-s001.docx]

**Supporting Information**

**Achieving High Stability and Capacity in Micron-sized Conversion-type Iron fluoride Li-metal Batteries**

Chiwon Choi^#1^, HM Yoon^#1^, Seungyeop Kang^2^, Dong Il, Kim^3^, John Hong^3^, Minjeong Shin^4^, Dong-Joo Yoo^2^, Minkyung Kim^*1^

^#^ The authors are equally contributed

^1^Department of Electronic Materials Engineering, Kwangwoon University, 60 Gwangun-ro 1-gil, Nowon-gu, Seoul, Republic of Korea 01897, Republic of Korea

^2^School of Mechanical Engineering, Korea University, 145 Anam-ro, Seongbuk-gu, Seoul 02841, Republic of Korea

^3^Department of Materials Science and Engineering, Kookmin University, Seoul, 02707, Republic of Korea

^4^School of Chemistry and Energy, Center for NanoBio Applied Technology, Sungshin Women’s University, 55 Dobong-ro 76 ga-gil, Gangbuk-gu, Seoul 01133, Republic of Korea

| **Element** | **Method** | **Result (Weight %)** | **Molar Ratio** |
| --- | --- | --- | --- |
| F-(Fluoride) | IC (Ion Chromatography) | 39.3 (%) | 2 |
| Fe (Iron) | ICP-OES (Inductively Coupled Plasma Optical Emission Spectrometry) | 58.6 (%) | 1 |
| C (Carbon) | Carbon & Sulfur Determinator | 0.328 (%) | Trace |

**Table S1.** Elemental composition of synthesized FeF_2_.

| **Parameter** | **Absorption (BJH)** | **Desorption (BJH)** |
| --- | --- | --- |
| **Cumulative Pore Volume (17 Å – 3000 Å)** | 0.026 cm^3^/g | 0.034462 cm^3^/g |
| **Average Pore Width (4V/A method)** | 45.636 Å | 46.273 Å |

**Table S2.** BJH results of synthesized FeF_2_.

| **Active Material**  **(Electrolyte)** | **Mass ratio (Active material: Acetylene Black: PVDF)** | **Current collector** | **Active material mass loading (mg/cm²)** |
| --- | --- | --- | --- |
| Synthesized FeF_2_  (EC/EMC) | 70:25:5 | Carbon-coated Al, Al | 1.95 (Carbon-coated Al), 1.0 (Al) |
| Synthesized FeF_2_ (G4/TTE) | 50:45:5 | Carbon-coated Al | 0.5-0.6 |
| Synthesized FeF_2_ (G4/TTE) | 70:25:5 | Carbon-coated Al | 1.6 |
| Commercial FeF_2_ (LHCEs, EC/EMC) | 70:25:5 | Al | 1.0-1.2 |

**Table S3.** Summary of Electrode Fabrication in this research.


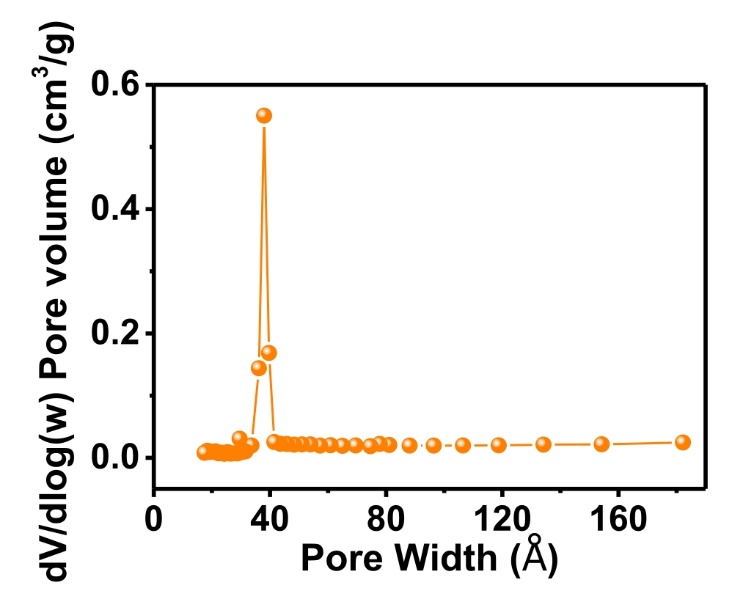


**Figure S1.** BJH desorption pore volume distribution


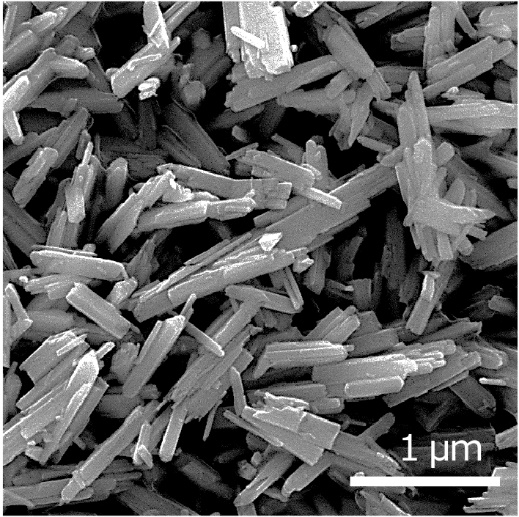


**Figure S2.** SEM image of iron precursor FeOOH.


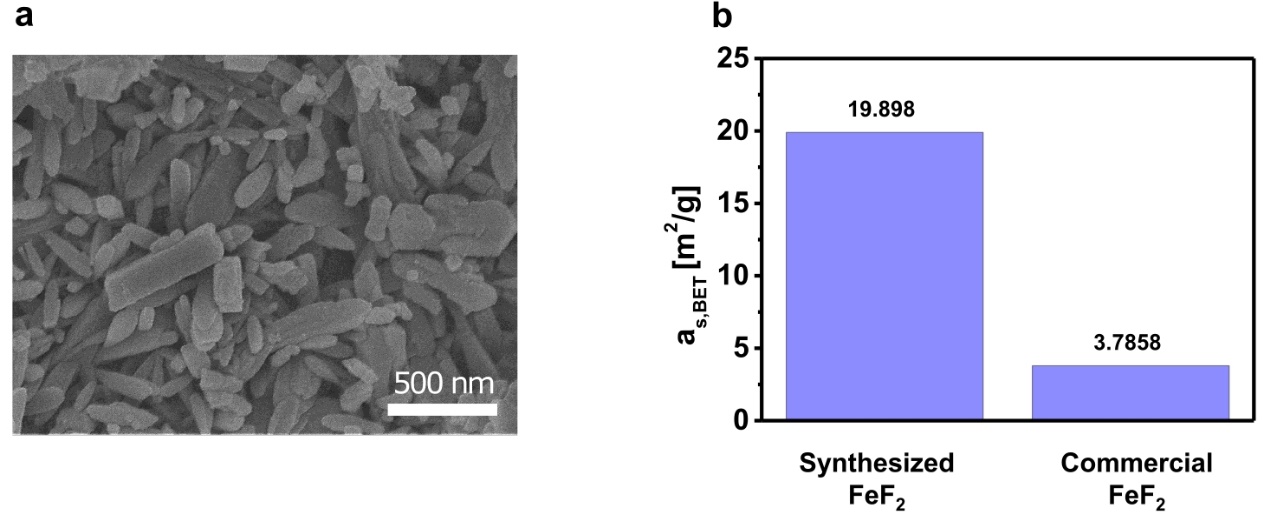


**Figure S3.** a) SEM image of commercial FeF_2_, b) BET results of synthesized FeF_2_ and commercial FeF_2_.

*
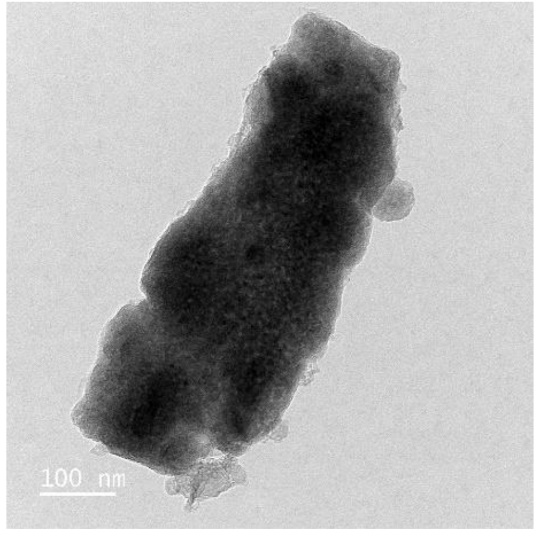
*

**Figure S4.** TEM image of FeF_2_ particle after 20 cycles.


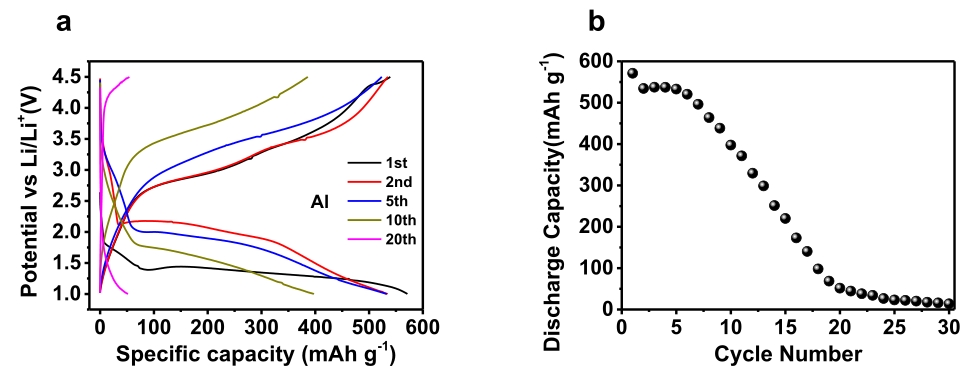


**Figure S5.** Electrochemical characteristics of FeF_2_ with Al current collector, a) Voltage profiles, b) Capacity retention.

**
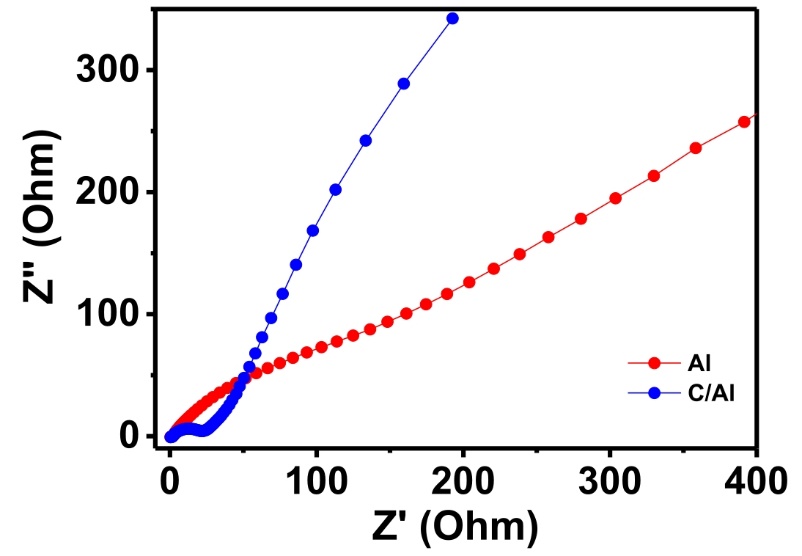
**

**Figure S6.** EIS results of Al and C/Al after 5 cycles.


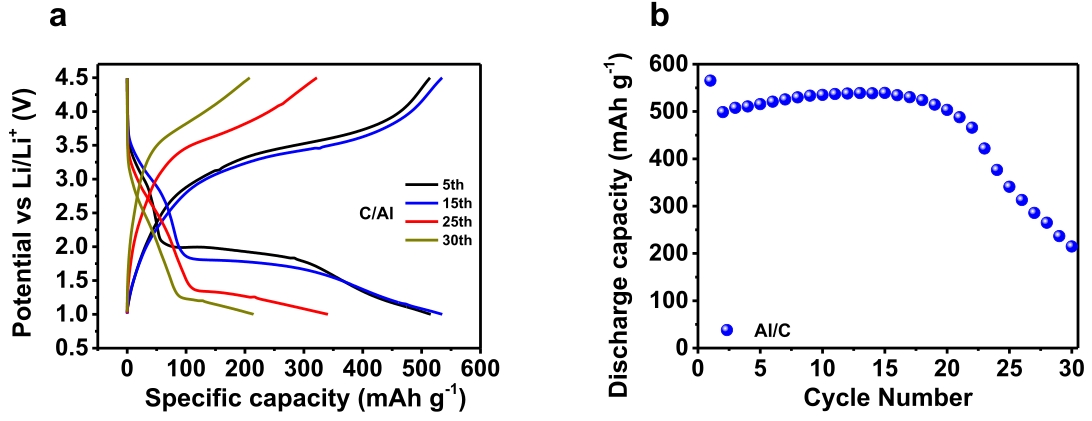


**Figure S7.** Electrochemical characteristics of FeF_2_ with carbon-coated Al current collector, a) Voltage profile, b) Cycle performance.


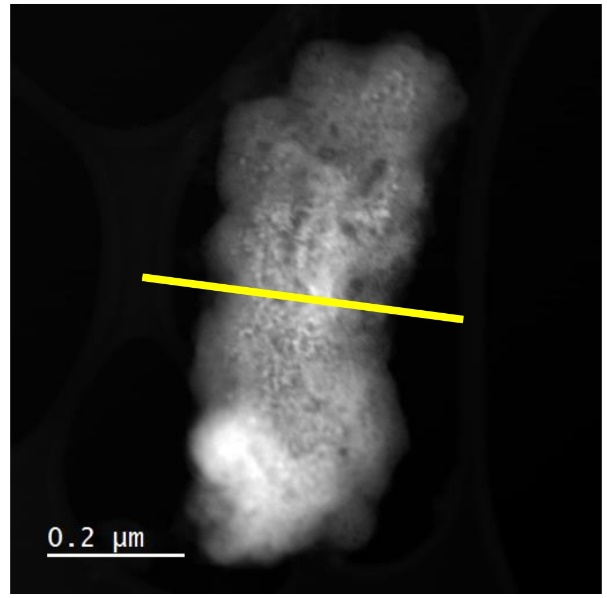


**Figure S8.** HAADF images of FeF_2_ particle after 25 cycles.


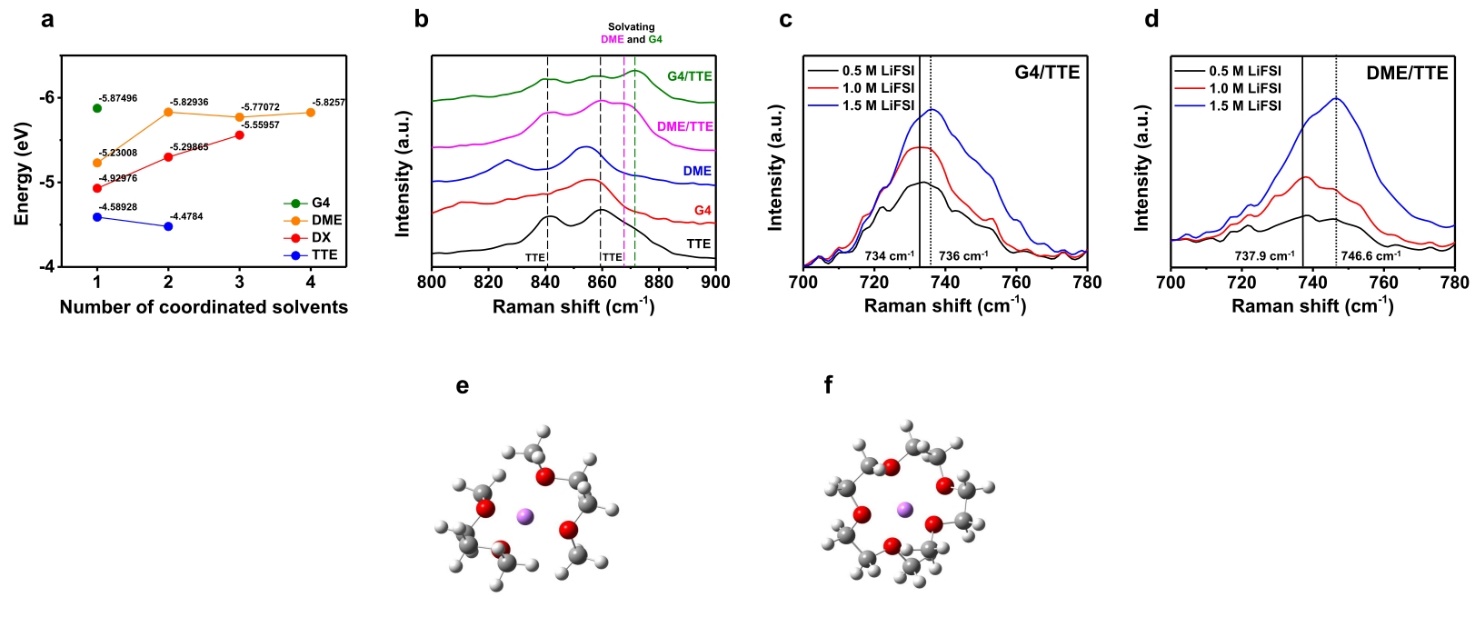


**Figure S9.** a) DFT calculation of binding energy of solvent with Li ion, b) Raman spectrum at 800-900 cm^-1^ of LHCEs, c) Raman spectrum at 700-780 cm^-1^ of G4/TTE, d) Raman spectrum at 700-780 cm^-1^ of DME/TTE, Solvation structure from DFT in e) Li-DME, f) Li-G4 (Pink: Li, Red: O, Gray: C, White: H).


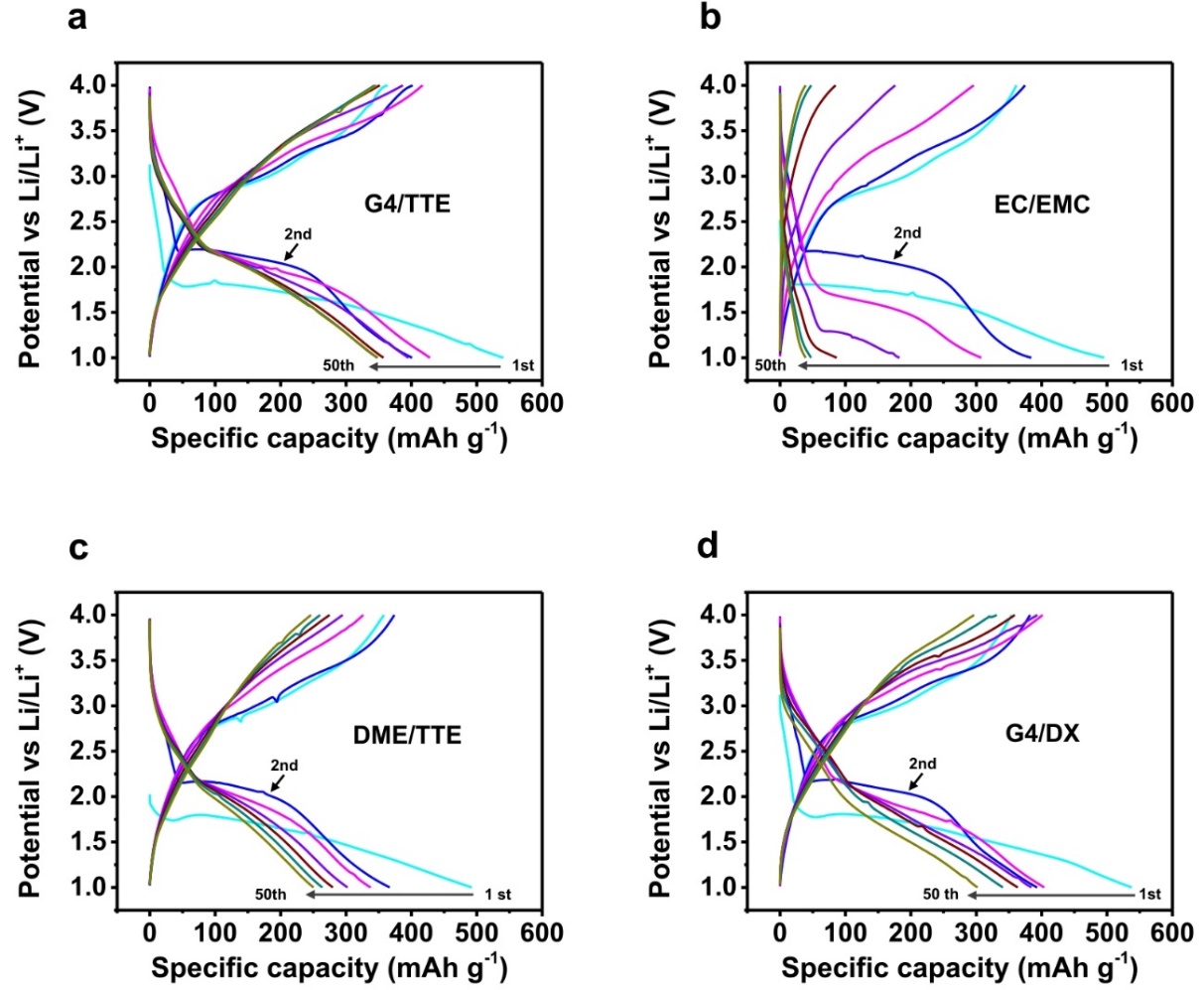


**Figure S10.** Voltage profiles of a) G4/TTE, b) EC/EMC, c) DME/TTE, d) G4/DX.


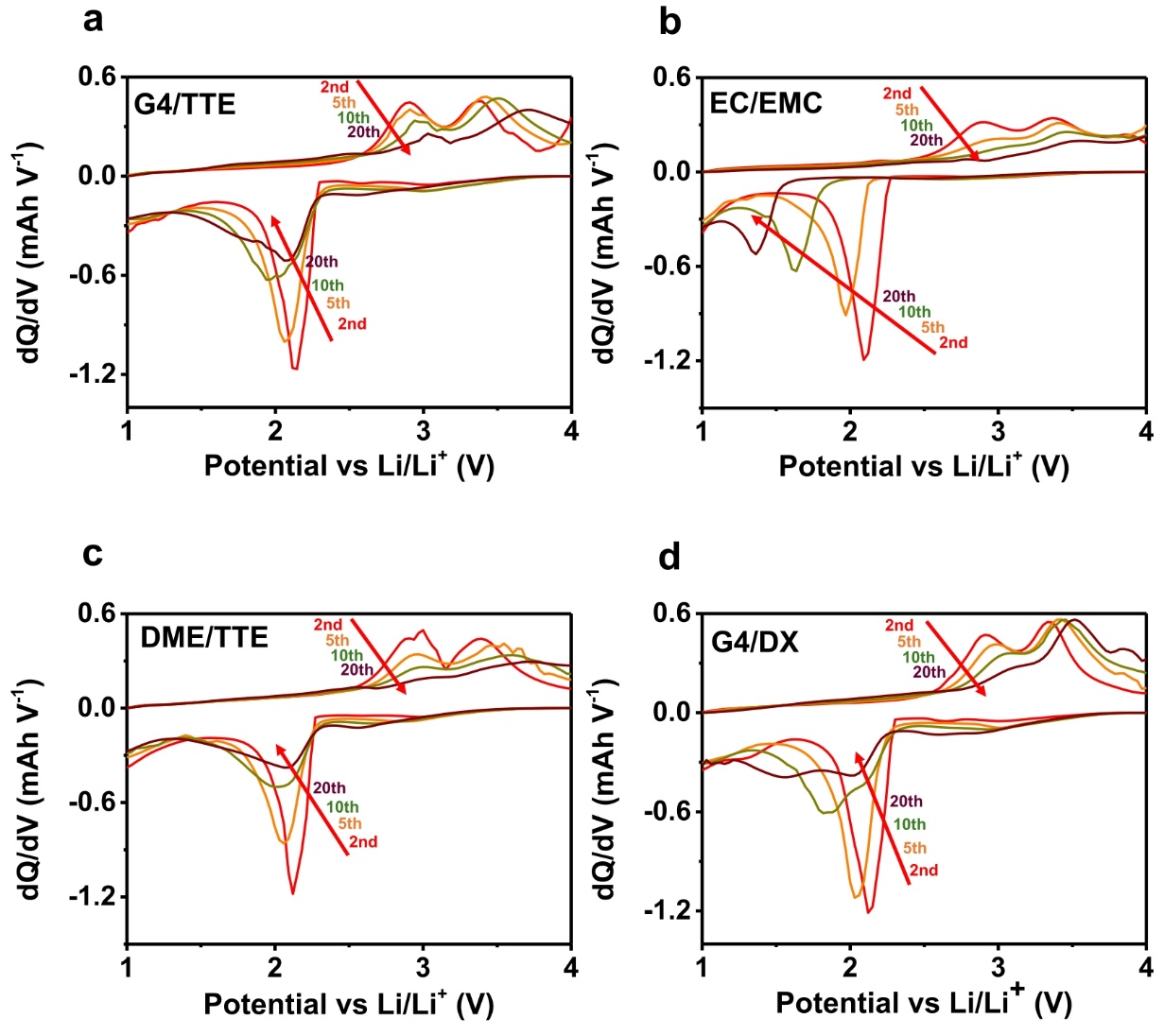


**Figure S11.** dQ/dV profiles of a) G4/TTE b) EC/EMC, c) DME/TTE, d) G4/DX.


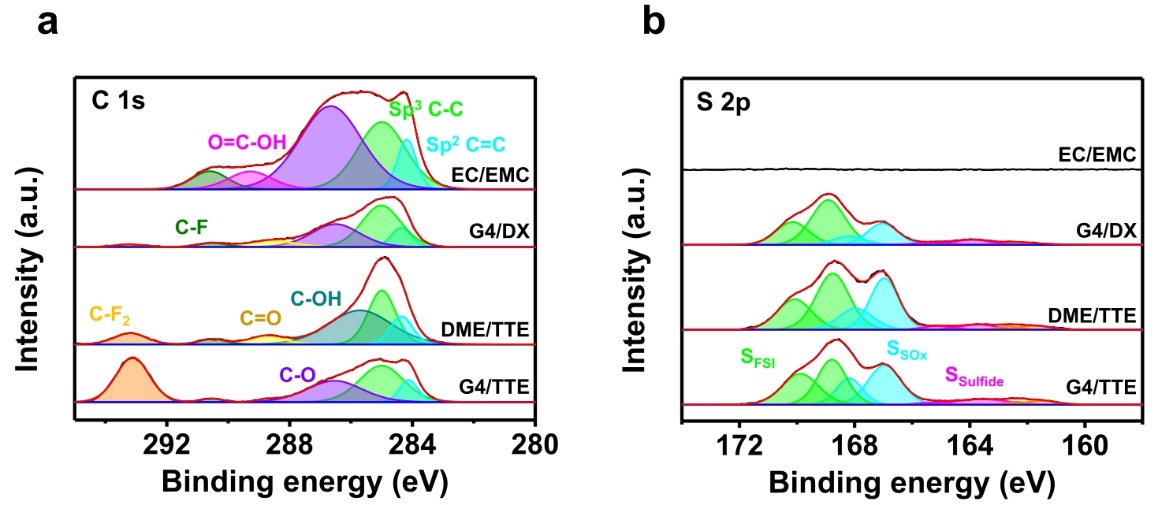


**Figure S12.** Investigation of surface of cycled FeF_2_ by XPS a) C 1s b) S 2p.

***
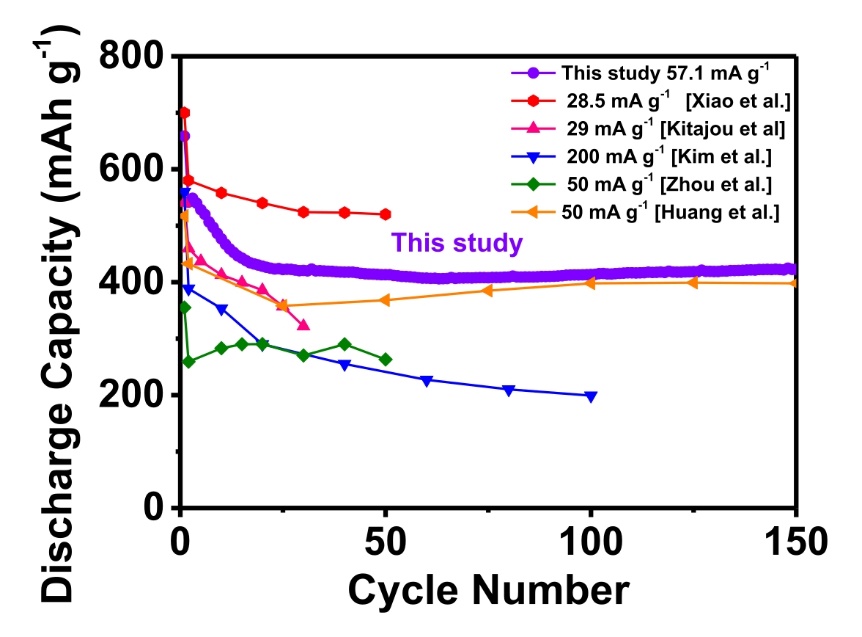
***

**Figure S13.** Comparison of the cycling stability of synthesized-FeF_2_ cathode with previous studies of similar FeF_2_ cathodes. (Xiao et al.^[1]^, Kitajou et al.^[2]^, Kim et al.^[3]^, Zhou et al.^[4]^, Huang et al.^[5]^)


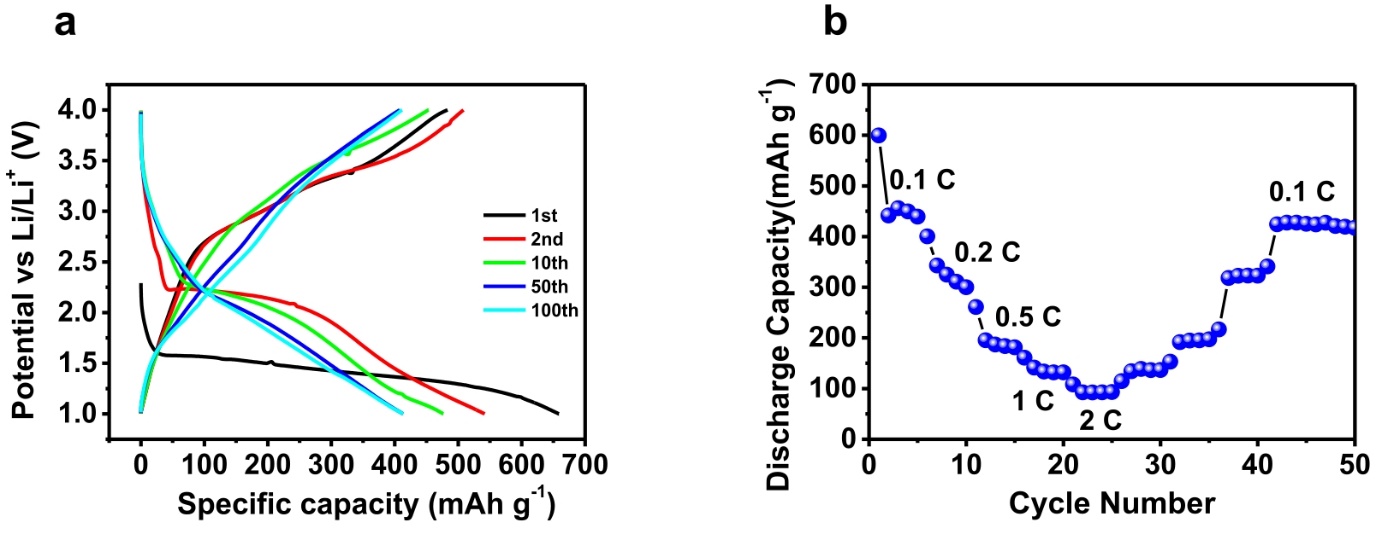


**Figure S14.** a) Voltage profiles of Li-synthesized FeF_2_ with G4/TTE at 0.1 C and 30 ℃, b) Rate capability of Li-synthesized FeF_2_ with G4/TTE.


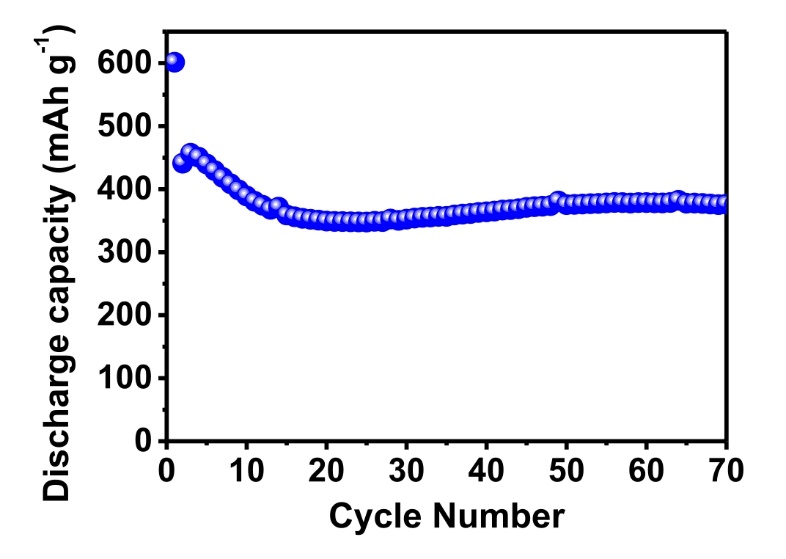


**Figure S15.** Discharge capacity per cycle of synthesized FeF_2_ (1.6 mg/cm^2^) with G4/TTE at 0.1 C and 30 °C

***
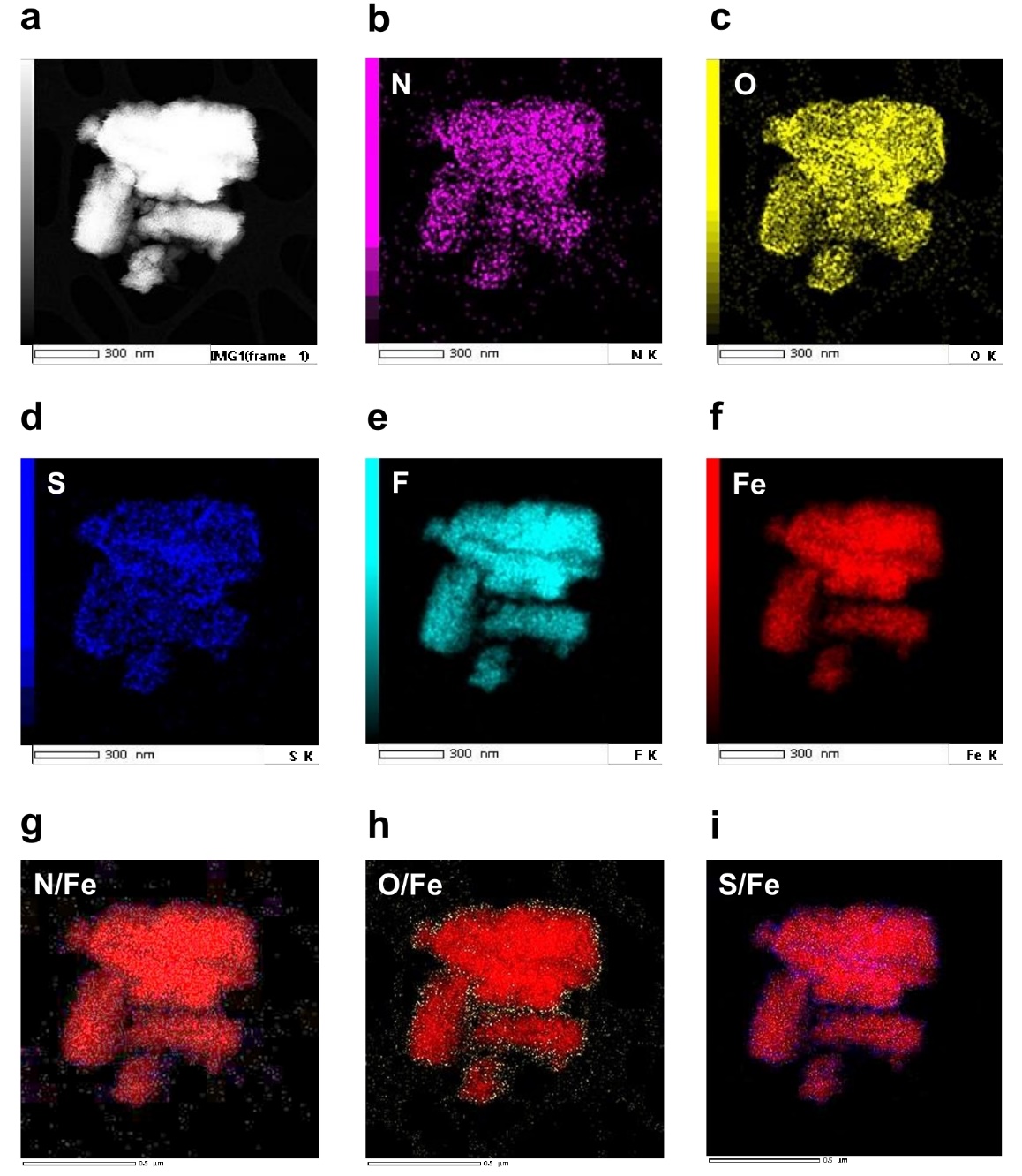
***

**Figure S16.** EDS mapping of post cycled synthesized FeF_2_ particles with G4/TTE. a) HADDF image of FeF_2_ particles. Elemental distribution of b) N, c) O, d) S, e) F, f) Fe, g) N with Fe, h) O with Fe, and i) S with Fe.

**REFERENCE**

[1] A. W. Xiao, H. J. Lee, I. Capone, A. Robertson, T. U. Wi, J. Fawdon, S. Wheeler, H. W. Lee, N. Grobert, M. Pasta, *Nat Mater* **2020**, *19*, 644-654. <https://doi.org/10.1038/s41563-020-0621-z>.

[2] A. Kitajou, H. Yamagishi, M. Katayama, K. Yoshii, M. Shikano, H. Sakaebe, S. Okada, *Journal of Electroanalytical Chemistry* **2022**, *920*, 116577. <https://doi.org/10.1016/j.jelechem.2022.116577>.

[3] S. Kim, J. Liu, K. Sun, J. Wang, S. J. Dillon, P. V. Braun, *Advanced Functional Materials* **2017**, *27*. <https://doi.org/10.1002/adfm.201702783>.

[4] J. Zhou, D. Zhang, X. Zhang, H. Song, X. Chen, *ACS Appl Mater Interfaces* **2014**, *6*, 21223-21229. <https://doi.org/10.1021/am506236n>.

[5] Q. Huang, K. Turcheniuk, X. Ren, A. Magasinski, A. Y. Song, Y. Xiao, D. Kim, G. Yushin, *Nat Mater* **2019**, *18*, 1343-1349. <https://doi.org/10.1038/s41563-019-0472-7>.
